# To Identify Myocardial Changes in Severely Malnourished Children: A Prospective Observational Study

**DOI:** 10.3389/fped.2015.00057

**Published:** 2015-09-03

**Authors:** Neeraj Kumar, Aakash Pandita, Deepak Sharma, Anita Kumari, Smita Pawar, Kishour Kumar Digra

**Affiliations:** ^1^Shyam Shah Medical College, Rewa, India; ^2^Department of Paediatrics, SMGS Hospital, Jammu, India; ^3^Department of Paediatrics, Pandit Bhagwat Dayal Sharma Post Graduate Institute of Medical Sciences, Rohtak, India; ^4^Nalanda Medical College and Hospital, Patna, India; ^5^Department of Obstetrics and Gynecology, Fernandez Hospital, Hyderabad, India

**Keywords:** malnutrition, body weight, height, BMI, ECG

## Abstract

**Aim and objective:**

The main purpose of this study is to identify the myocardial changes in severely malnourished children.

**Materials and methods:**

This prospective, observational study, conducted for a period of 1 year, enrolled 200 children (120 males and 80 females) between 6 months and 5 years of age with severe protein–energy malnutrition, according to the criteria of the World Health Organization. The parents were duly informed, the study was explained and written consent was obtained. A random selection of cases was carried out, and they were further divided into five groups according to their age as follows: <1, 1–2, 2–3, 3–4, and 4–5 years. Electrocardiograms (ECGs) were taken at the time of admission for all the cases and the control group and were taken again after nutritional therapy either at the time of discharge or after a fortnight. The differences were then compared.

**Results:**

On admission, 32% of cases had flat P-wave, out of which 75% reverted to normal with therapy. Similarly, 84% of cases had increased corrected QT interval at the time of admission. ST segment was depressed only in 8% of cases. 88% of cases had altered (flat to depressed) T wave at the time of admission. With the help of nutritional supplementation, all these abnormalities were back to a normal level at the time of discharge.

**Conclusion:**

Electrocardiographic changes may be of help in assessing the severity and prognosis of severe acute malnutrition. The reversibility of ECG changes with dietary treatment suggests that the cardiac changes are not permanent in nature and may not affect adult life if the malnutrition is corrected. The cardiac status as denoted by heart rate remained the same even after a fortnight, suggesting that prolonged therapy and assessment of cardiac status is warranted even after fortnight therapy.

## Introduction

Protein–energy malnutrition (PEM) affects tissue protein throughout the body and causes pronounced wasting of skeletal muscles. In view of this, it is surprising that little attention has been paid to the probability of getting the heart muscle affected and the possibility of compromising the cardiac functions. By the general atrophy of all muscular tissues, the heart also gets affected but less severely. Although the heart is usually normal or small in size radiologically, cardiomegaly has been found in marasmic infants (1). On the contrary, the heart is underweight if it is kwashiorkor. Early literature describes the appearance of the heart in wasting diseases as having “brown atrophy" with emphasis on the smallness of heart shadow on X-rays. Other changes include myocardial fiber atrophy with variations in fiber size, vacuolations within the cells, fading of the striations, and changes in nuclei. These changes are mostly found in the left ventricle and the conducting tissue. All these pathological changes lead to disturbed cardiac functions in the electrical activity of the heart that may be reflected in the electrocardiogram (ECG) (2, 3). However, studies on electrocardiographic changes in PEM are scant. The reduction of amplitude of all the waves is the most striking change in all the leads, especially in kwashiorkor. Both tachycardia and bradycardia of sinus origin have been reported. To a great extent, PEM is one of the leading causes of mortality and morbidity in the pediatric population in developing countries (4). It is prevalent in the Rewa district of India having a high incidence of low birth weight babies, low literacy rate, and poverty. There are still gaps in our knowledge regarding protein energy malnutrition especially with regard to cardiac functions. An attempt has been made in this study to analyze the cases of severe PEM, especially with regard to electrocardiographic changes, and to correlate these changes in relation to the clinical picture and cardiac status.

## Materials and Methods

This was a prospective, observational study done in the Department of Paediatrics, Shyam Shah Medical College associated with Gandhi Memorial Hospital, Rewa (Madhya Pradesh, India), for a period of 1 year. The study sample consisted of 200 cases (120 male children and 80 female children, who were admitted during the study period of 1 year) of severe PEM according to the criteria of the World Health Organization (WHO). All the cases were divided into five groups according to their age as follows: <1, 1–2, 2–3, 3–4, and 4–5 years. A random selection of cases was done irrespective of caste, creed, religion, sex, and socioeconomic status. The study was approved by the institutional ethical committee and the institutional research board, and written consent was obtained from the caretakers or parents of the children before enrollment. A short history about febrile illnesses, burning micturition, cough, and dyspnea/breathlessness was also recorded. A complete general physical examination from head to toe was done for all the enrolled children. Vital parameters and temperature were also recorded. The children were clinically assessed for anemia, jaundice, cyanosis, clubbing, lymphadenopathy, and edema and congenital anomalies were ruled out. A detailed systemic examination of all systems (respiratory system, cardio-vascular system, gastro-intestinal system, and nervous system) was carried out in order to exclude systemic disorders such as congenital heart disease, renal disorders and liver diseases.

### Selection criteria

The selection criteria for the study are as follows:

1. Children must be between 6 months and 5 years of age (who were selected from inpatients).
2. Children must also fulfill the WHO criteria for severe malnutrition.

The WHO criteria for severe malnutrition are as follows:

- Severe wasting (weight for length < 70%);
- Bilateral noninflammatory symmetrical pitting edema of feet;
- Visible severe wasting; and
- Mid-upper arm circumference (MUAC) < 11.5 cm.

Exclusion criteria are as follows:

1. Known metabolic disorders;
2. Clinically suspected endocrine disorders;
3. Known connective tissue disorders in a mother (e.g., systemic lupus erythematosus (SLE));
4. Any congenital malformation (e.g., cleft lip and cleft palate); and
5. Age <6 months or > 5 years.

The parents were duly informed and explained about the study, and written consent was obtained.

### Methods

For all the admitted children below 5 years of age, anthropometric assessment was done, and those who fulfilled the WHO criteria for severe malnutrition were enrolled in the study. None of the cases suffered from cardiac disease (congenital/acquired), known metabolic disorders, clinically suspected endocrine disorders, or known connective tissue disorders in a mother (e.g., SLE), which could affect the readings of ECG. Cases were recorded in a pretested and predesigned *pro forma*.

### Anthropometry

#### Weight

Children of <10 kg body weight were weighed on an electronic weighing machine with an accuracy of + 5 g, and children of >10 kg were weighed on Beam Scale or Salter-type scale. Double weighing was carried out for restless children; first the mother was weighed separately, then she was weighed holding her child and the difference was calculated.

#### Length

An infantometer was used for measuring the length of the children who were below 2 years of age. For children above 2 years of age, a vertical measuring rod or stadiometer was used.

#### Weight for length

For the comparison of weight and height, the chart of the United Nations Children’s Fund was used.

#### Assessment of MUAC

To assess the nutritional status of the children, MUAC measurement was taken on the left arm midway between the acromion and the olecranon process by a flexible and non-stretchable measuring tape that is not affected by temperature.

### Collection of data

#### History

After explaining the study to the parents, for all cases a detailed history regarding the presenting illness was examined for corroborative evidence indicating the precipitation of malnutrition. Presenting complaints, such as diarrhea, dysentery, vomiting, cough, fever, and shortness of breath, were explored in detail. The immunization status and prior measles or pertussis infections were also recorded. Socioeconomic status of the family was ascertained using the Modified Kuppuswamy’s Socioeconomic Scale.

### Clinical examination

A general physical examination was carried out to look for pallor, edema, wasting, icterus, lymphadenopathy, hair and skin changes, mental changes, and signs of vitamin A deficiency. Detailed systemic examination was performed, and children were also examined for any congenital malformation.

### Laboratory investigations

Hemoglobin estimation and chest X-ray were performed for all the admitted patients.

Electrocardiographic changes were studied at the time of both admission and discharge.

### The control group

The control group, consisting of healthy children of comparable age were selected from the siblings of patients admitted with a different disease (not malnutrition).

### Nutritional advice

Mothers were educated about the requirements of their children and the ways to prepare energetic and nutrient-dense food. They were advised to feed their children every 4 h with a total of 5–6 feeds a day. Parents were told to offer their children an amount of food, such that some food was left in the pot after the child had eaten. Mothers were counseled in the local language and the dietary advice provided was given with consideration to the parents’ economic capacity. Parents were also educated on good hygiene practice, how to prevent malnutrition from recurring, the treatment of diarrhoea with oral rehydration salts and the recommended immunization schedule for their children. After being given all this information, they were told that ECG would be taken again at the time of discharge.

The patients were also given ready-to-use therapeutic food (RUTF) in addition to their nutritional supplementation which is rich in proteins and calories.

The simplest recipe for RUTF is the one that has only two ingredients, such as, a cereal or root mixed with a legume. However, other foods must be added to this basic mix in order to make it a multi-mix that is nutritionally suitable for the treatment of acute malnutrition (Table 1).

**Table 1:** **Ingredients for RUTF**.

Ingredients	Weight (%)
Milk powder	30
Sugar	28
Vegetable oil	15
Peanut butter	25
Mineral vitamin mix	1.6

*Peanut butter is simply peanuts that have been roasted and ground without adding oil, salt, or preservatives*.

A nutritionally suitable multi-mix of RUTF has four basic ingredients as follows:

1. A staple as the main ingredient – preferably a cereal;
2. A protein supplement from a plant or animal food – beans, groundnuts, milk, meat, chicken, fish, eggs, etc.
  To be practical, such foods must be low cost. Thus, legumes and oilseed are preferred as they are cheaper than milk products or other animal products.
3. A supplement with vitamin and mineral – vegetables and/or fruits; and
4. An energetic supplement – fat, oil, or sugar to increase the energetic concentration of the mix.

In addition, an ideal RUTF formulation must have the following attributes:

- Good nutritional quality (i.e., protein, energy, and micronutrient content);
- Long shelf life;
- Palatable to children;
- Consistency and texture that are suitable for feeding the children;
- No additional processing prior to feeding;
- Amino acid complementation for maximum protein quality;
- Product stability; and
- Easy access of the ingredients in developing countries.

As the name implies, RUTF does not need any preparation before consumption, making it very feasible where fuel and facilities for cooking are limiting constraints. RUTF has a very low water activity; thus, it is impossible for significant bacterial growth to occur in these foods. This allows locally produced RUTF to be safely stored at ambient tropical conditions for 3–4 months. RUTF has a very high energy density of about 23 kJ/gm (5.5 kCal/g). A severely malnourished child needs to consume just a few spoonfuls of RUTF 5–7 times a day to achieve sufficient nutrient intake for complete recovery. RUTF must be consumed with water, and no other foods are necessary for the rehabilitation of the malnourished child.

The signs of clinical improvement taken into account were as follows:

- Increase in weight at the time of discharge;
- Increase in appetite at the time of discharge;
- Decrease in edema after nutritional rehabilitation at the time of discharge; and
- Normalization of altered mental changes at the time of discharge.

### Electrocardiography

Electrocardiograms were recorded for all the cases and the control group in supine position using standard limb leads (i.e., I, II, and III), unipolar limb leads (i.e., avR, avL, and avF), and unipolar precordial leads (i.e., V1, V2, V3, V4, V5, and V6). Electrocardiogram was taken before starting the treatment and at the time of admission and was taken again at the time of discharge or after 14 days of treatment with a diet rich in proteins and calories. The differences were then compared.

Electrocardiograms were recorded with BPL Cardiart 6208 at a paper speed of 25 mm/s. The record was standardized in every lead to give 10 mm deflection with the introduction of 1 mV current into the circuit (i.e., 1 mV = 10 mm). Infants and children were laid in the supine position, and all the records were taken when they were asleep after feeding or after a preliminary bed rest. Unipolar precordial leads were recorded from V1 to V6 using Wilson’s central terminal as the indifferent electrode. The precordial positions were localized with great care as per the recommendations of the British Cardiac Society (1949). Enough care was exercised when applying and removing the excess of electrode jelly.

The smallest unit of measurement was 0.5 mm (0.05 mV). The following parameters were studied:

1. Heart rate (HR);
2. Duration and amplitude of P wave
3. PR interval;
4. Duration and amplitude of QRS;
5. Corrected QT (QTc) interval;
6. ST segment;
7. T wave; and
8. Axis.

The electrical axis in the standard frontal plane was determined by using the triaxial system of Bayley. When the cycle length varied by more than 10% in any given strip of ECG, the record was said to display sinus arrhythmia. QTc interval was measured only in standard limb lead II, unless there was a significant difference between this lead and either lead I or lead III due to an isoelectric Q wave or flat T wave in lead II. Measurements were taken in three cardiac cycles by always including the shortest and the longest cycle lengths available. From these measurements, the average QT interval and RR interval were then derived. Then, the QTc interval was determined for all the cases in the nomogram representation. The P wave and QRS measurements were also taken in standard lead II. For the abnormalities of T wave, measurements were taken in V5 or V6. Axis was calculated from the standard hexaxial reference system. The amplitude of any positive deflections, such as P, R, and T waves, was measured from the upper margin of the baseline to the very top of the positive deflections. The amplitude of any negative deflections was measured from the lower margin of the baseline to the lowest point of wave.

The normal parameters of ECG for the patients taken were HR, 65–120/min (6 months–5 years); PR interval, 0.12–0.20 s; QRS interval, <0.12 s; QTc interval, <0.44 s; and axis, −30° to 90°. Normal amplitudes of different waves were taken as: P-wave >1.5 mm (normal); P-wave <1.5 mm (flattened); ST segment below the isoelectric line (depressed); T wave in V5 or V6 below the isoelectric line (depressed); T wave <2 mm above the isoelectric line (flattened).

*P* (probability) value was calculated from chi-square table and degrees of freedom (DF).

## Results

### Heart rate

Table 2 shows that both tachycardia and bradycardia of sinus origin were present in few of the cases (normal HR being 65–120/min in the age group of 6 months–5 years), whereas they were normal in the control group. The value of chi-square was 1.011 and the expected value for DF was 2, so *P* = 0.6033; therefore, the finding is not significant.

**Table 2:** **Distribution of study cohort according to Heart Rate**.

Heart Rate	Cases		Control group	
	Admission	Discharge	Admission	Discharge
<100	12	12	24	–
100–150	184	188	36	–
>150	04	00	–	–

### P wave

Table 3 shows that 32% of the cases had flat P wave at the time of admission, of which 24% were back to normal at the time of discharge after nutritional supplementation. On the other hand, all the children in the control group had normal P wave. This indicates that there was a decrease in amplitude of P wave in few cases due to malnutrition, which may be corrected with nutritional supplementation. The value of chi-square was 9.00, and the expected value for DF was 1, so *P* < 0.05. Therefore, the finding is statistically significant.

**Table 3:** **Distribution of study cohort according to the changes in P wave**.

P wave	Cases		Control group
	Admission	Discharge	
Normal	136	184	60 (Normal)
Flat	64	16	
Peak	–	–	

### PR interval, QRS complex, and axis

No abnormalities in PR interval, QRS complex, and cardiac axis were seen in either group.

### QTc interval

Table 4 shows that 84% of the cases had an increased QTc interval at the time of admission, of which the majority had a normal QTc interval at the time of discharge after nutritional supplementation, which indicates that severe malnutrition has an association with QTc interval. All the children in the control group had normal QTc interval. The value of chi-square was 54.95, and the expected value for DF was 1, so *P* < 0.0001. Therefore, the finding is statistically significant.

**Table 4:** **Distribution of study cohort according to the changes in QTc interval**.

QTc interval	Cases		Control group
	Admission	Discharge	
Normal	32	180	60 (Normal)
Increased	168	20	

### ST segment

In the present study, the ST segment was depressed in 8% of the cases, where the cases became normal again after nutritional rehabilitation (Table 5). This could be considered as the variant of normal. However, ST segment was normal in the control group. The value of chi-square was 54.95, and the expected value for DF was 1, so *P* < 0.05. Therefore, the finding is statistically significant.

**Table 5:** **Distribution of study cohort according to the changes in ST segment**.

ST changes	Cases		Control group
	Admission	Discharge	
Normal	184	200	60 (Normal)
Depressed	16	00	
Elevated	00	00	

### T wave

Table 6 shows that 88% of the cases had altered (flat to depressed) T wave at the time of admission, of which a majority of them came back to normal after nutritional supplementation at the time of discharge. This indicates an alteration in repolarization due to malnutrition. None of the children in the control group had an abnormal morphology of T wave. The value of chi-square was 57.936, and the expected value for DF was 2, so *P* < 0.0001. Therefore, the finding is statistically significant.

**Table 6:** **Distribution of study cohort according to the changes in T wave**.

T wave	Cases		Control group
	Admission	Discharge	
Normal	24	176	15 (Normal)
Depressed	112	12	
Elevated	00	00	
Flat	64	12	

### Relation of weight/height with QTc interval

Table 7 shows that 124 cases (86%) with weight/height <3^rd^ percentile had increased QTc intervals (>0.5 s in majority of the cases), whereas 20 cases (14%) had QTc intervals of >0.44–0.50 s. The 124 cases (86%) became normal at the time of discharge after dietary supplementation rich in proteins and calories. In the group with weight/height between 3^rd^ and 50^th^ percentile, increased QTc interval was present in all the 24 cases (but it was between 0.44 and 0.5 s). In all the 24 cases, QTc interval became normal at the time of discharge after dietary supplementation. From these variables, it is clear that the degree of QTc interval prolongation was more in <3^rd^ percentile (176) group than the 3^rd^–50^th^ percentile (24) group, and a majority of patients in both the groups had a normal QTc interval after dietary supplementation rich in proteins and calories and RUTF. The value of chi-square was 94.345, and the expected value for DF was 6, so *P* < 0.0001. Therefore, the finding is statistically significant.

**Table 7:** **Distribution of study cohort demonstrating relation of weight/height with QTc interval**.

QTc interval	Weight/height		Weight/height		Control group
	<3d^rd^ percentile (176)		3–50h^th^ percentile (24)	
	Admission	Discharge	Admission	Discharge	
<0.44 s (Normal)	32	156	00	24	60 (Normal)
**Increased**
A. >0.44–0.50 s	20	20	24	00	
B. >0.50 s	124	00	00	00	

## Discussion

The present study was undertaken for observing electrocardiographic changes in the children with severe acute malnutrition (SAM), which included a total of 200 cases (120 males and 80 females) in the age group of 6 months–5 years. A control group of a total of 60 healthy children (28 males and 32 females), matched for age and sex, were also studied. It was extremely difficult to recruit healthy children for the control group who fit the criteria of normal. This reflects the great prevalence of mild and moderate PEM in this area of Rewa and the reasons for the inclusion of a smaller number of children in the control group. None of the cases suffered from cardiac disease (congenital/acquired), known metabolic disorders, clinically suspected endocrine disorders, or known connective tissue disorders in a mother (e.g., SLE), which could produce electrocardiographic changes. Electrocardiograms were taken on admission to the hospital, before starting the treatment in all cases. Electrocardiograms were then repeated at discharge following treatment with a diet rich in protein, calories and RUTF. The cases where ECGs could not be taken due to any reason were excluded from this study. The siblings of the patients admitted with other diseases (other than malnutrition) who were apparently normal were chosen for the control group.

### Electrocardiographic findings

The results reported here are generally in line with the observations of other researchers with few alterations.

#### Heart Rate

In the present study, the HR ranged from 88 to 156, and the *P* value is >0.05, which is insignificant (Table 2). For 4 cases, notable tachycardia was present with HR as high as 156, and 12 cases showed bradycardia. Both tachycardia and bradycardia have been described by previous researchers.

In the 50 cases, in whom ECG was taken again after the treatment with a diet rich in proteins and calories and RUTF, no significant change in HR was observed. In one case, HR decreased from 156 to 128, but there is no significant correlation reported between the malnutrition and HR in the studies (5, 6).

There was no correlation between the HR and clinical severity too. A factor that had to be taken into account when assessing the HR was the presence of anemia of varying severity in all the cases. It was, however, found that there was no correlation between the severity of anemia and the HR. To some extent, the significance was vitiated by the fact that it was not always possible to keep them free from excitement.

#### The Rhythm

Out of 200 cases studied, normal sinus rhythm was observed in 184 cases, whereas 16 cases showed sinus arrhythmia. It is, however, not enough to draw conclusion from the presence of these arrhythmias, considering the fact that they may sometimes be observed in normal subjects. All these irregularities of sinus rhythm disappeared in the records obtained after the treatment.

Regular sinus rhythm has been observed by previous researchers in accordance with our study (5–7).

### Interval changes

#### PR Interval

In the present study, there was no significant change in the PR interval of the patients compared with the control group. There is paucity of data regarding the variation in PR interval with malnutrition (5–7).

#### QRS Duration

No major study has reported any change in the QRS duration. In the present study, QRS duration was normal compared with the control group. No change in QRS duration was noticed after the treatment (5–7).

#### QTc Interval

In the present study, there was an increased QTc interval for most of the cases that was statistically highly significant. After nutritional supplementation, QTc interval came back to normal for majority of the cases (Table 4).

An increased QTc interval has been reported by most other researchers (5–10). The occurrence of prolonged QTc interval relative to the cycle length in almost all cases and its resuming to normal by the therapy with a diet rich in proteins and calories and RUTF seems significant. In the present study (Table 4), there was an increase in QTc interval in almost all cases of SAM with grading from <3 SD (>0.5 s) to 3–50 SD (0.44–0.5) of weight/height that was statistically highly significant. The same was true for T wave morphology. Depressed/flattened T wave at the time of admission was seen in both the groups of weight/height, i.e., <3 SD and 3–50 SD, and this became normal after the therapy and with RUTF. These changes can be attributed to the low potassium and calcium found in malnourished children.

### Morphological (amplitude) changes

#### P Wave Amplitude

In the present study, the amplitude of P wave was flat in 32% of the cases, and this decrease was statistically significant. A majority of the cases (75%) became normal after nutritional rehabilitation (Table 3). There is paucity of data in relation to P wave with the degree of malnutrition.

#### T Wave Amplitude

The T wave also showed markedly diminished amplitude in majority of the cases. T wave was depressed in 56% of the cases, whereas it was flat in 32% of the cases. This decrease was statistically significant (Table 6). After the treatment with a diet rich in proteins and calories, there was a marked increase in the amplitudes of the deflections in all the leads practically for all the cases. The T waves that were isoelectric or depressed before treatment became erect after treatment. The significance of T wave in malnourished children is not reported in the studies (9, 10).

#### ST Segment

In the present study, the ST segment was depressed only in 8% of the cases, where it became normal for them after nutritional rehabilitation. These changes were present in only few cases and can be considered as a variant of normal (Table 5). There is paucity of data in the change of ST segment with malnutrition.

#### QRS Axis

In the present study, QRS axis in frontal plane was within normal limits in all the cases at the time of both admission and discharge.

In the present study, there was increase in QTc interval and decrease in amplitude of waves of all the cases at the time of admission. There was remarkable improvement in clinical and ECG changes after administering a diet rich in proteins and calories and with RUTF.

## Conclusion

The results observed after the analysis of records were in line with the observations made by most other researchers with some differences. Analysis of the records revealed the following:

1. There was no significant change in HR with nutritional therapy in the cases.
2. In most cases of SAM, there was a regular sinus rhythm. Occasional cases showed arrhythmias.
3. PR interval was normal in all the cases.
4. QTc interval was significantly increased in majority of the cases (prolongation was more in weight/height group <3^rd^ percentile than in the group with 3^rd^–50^th^ percentile). The interval became normal in most of the cases after therapy with a diet rich in proteins and calories and RUTF.
5. The amplitude of all deflections (P wave, QRS complex, and T wave) in all leads was subnormal in majority of the cases. After treatment with a diet rich in proteins and calories and RUTF, there was an increase in the amplitude of all deflections in all leads.
6. The increase in T wave amplitude paralleled the clinical improvement. The T wave that was isoelectric or depressed at the time of admission became erect after the treatment.
7. ST segment did not show any significant change.
8. QRS interval was normal in all the cases.
9. QRS axis was normal in all the cases.

Electrocardiographic changes may be of help in assessing the severity and prognosis of SAM. The reversibility of ECG changes with dietary therapy suggests that the cardiac changes are not permanent in nature and may not have a long-term effect if malnutrition is corrected. The cardiac status as denoted by heart rate remained the same even after a fortnight, suggesting that prolonged therapy and assessment of cardiac status is warranted even after fortnight therapy.

## Author Contributions

NK and AK wrote the first draft of the manuscript. DS, AK, SP, KKD and AP helped in writing the manuscript and did primary corrections in the manuscript. AP, SP and KKD made final corrections to the manuscript before submission. There was no honorarium, grant, or other form of payment given to anyone to produce the manuscript. All the authors approved the submission of this version of the manuscript and take full responsibility for the manuscript.

## Conflict of Interest Statement

The authors declare that the research was conducted in the absence of any commercial or financial relationships that could be construed as a potential conflict of interest. There are no prior publications or submissions with any overlapping information, including studies and patients.
